# Ubiquitination of Major Histocompatibility Complex II Changes Its Immunological Recognition Structure

**DOI:** 10.3390/ijms242317083

**Published:** 2023-12-03

**Authors:** Yuko Kozono, Masahiro Kuramochi, Yuji C. Sasaki, Haruo Kozono

**Affiliations:** 1 Research Institute for Biomedical Sciences, Tokyo University of Sciences, Noda 278-0022, Chiba, Japan; y-kozono@rs.tus.ac.jp; 2 Graduate School of Science and Engineering, Ibaraki University, Hitachi 316-0033, Ibaraki, Japan; masahiro.kuramochi.vw26@vc.ibaraki.ac.jp; 3 Department of Advanced Material Science, Graduate School for Frontier Sciences, The University of Tokyo, Kashiwa 277-8568, Chiba, Japan; ycsasaki@edu.k.u-tokyo.ac.jp; 4 AIST-U Tokyo Advanced Operando Measurement Technology Innovation Laboratory (OPERANDO-OIL), National Institute of Advanced Industrial Science and Technology (AIST), 6-2-3 Kashiwanoha, Kashiwa 277-0882, Chiba, Japan; 5 Japan Synchrotron Radiation Research Institute, SPring-8, 1-1-1 Kouto, Sayo 679-5198, Hyogo, Japan

**Keywords:** ubiquitin ligase, MHC, dendritic cells, LAG-3, single-molecule biophysics

## Abstract

Ubiquitination is a process that dictates the lifespan of major histocompatibility complex class II (MHC II)/peptide complexes on antigen-presenting cells. This process is tightly controlled by the levels of ubiquitin ligases, and disruptions in the turnover of MHC II can lead to the improper development of CD4+ T cells within the thymus and hinder the formation of regulatory T cells in the peripheral tissue. To investigate the underlying mechanisms, we utilized dendritic cells lacking the Membrane-associated RING-CH (MARCH) I ubiquitin ligase. We discovered that the overexpression of MARCH I decreases the interaction with LAG-3. Moreover, the MHC II molecules tethered with ubiquitin also showed diminished binding to LAG-3. We employed Diffracted X-ray Blinking (DXB), a technique used for single-molecule X-ray imaging, to observe the protein movements on live cells in real time. Our observations indicated that the normal MHC II molecules moved more rapidly across the cell surface compared to those on the MARCH I-deficient dendritic cells or MHC II KR mutants, which is likely a result of ubiquitination. These findings suggest that the signaling from ubiquitinated MHC II to the T cell receptor differs from the non-ubiquitinated forms. It appears that ubiquitinated MHC II might not be quickly internalized, but rather presents antigens to the T cells, leading to a range of significant immunological responses.

## 1. Introduction

Major histocompatibility complex class II (MHC II) molecules play a crucial role in presenting external antigens (Ag) to CD4+ T cells [1] and triggering an acquired immune response with a tuned specificity. Antigen-presenting cells (APCs) with robust endocytosis mechanisms capture and digest microbial antigens, loading the resulting peptides onto MHC II within late endosomes/lysosomes. This function is not restricted to the APCs; epithelial and endothelial cells also express MHC II when infected or inflamed [2]. Additionally, MHC II can present self-derived peptides, which can lead to the development of regulatory T cells and influence T cell selection within the thymus [3]. The balance of MHC II expression is delicately controlled by the cytokines and the process of ubiquitination.

Newly created MHC II molecules initially contain class II-associated invariant chain peptides (CLIP) in their peptide-binding groove, which are subsequently displaced by locally generated peptides in the presence of the molecule DM. Following this, the complex becomes tagged with ubiquitin via the action of the enzyme Membrane-associated RING-CH (MARCH) I [4]. High levels of MARCH I expression lead to a decrease in MHC II molecules on the cell surface. Conversely, B cells lacking MARCH I exhibit an increase in the MHC II surface levels [5]. Immature dendritic cells exhibit the increased surface expression of MARCH I. However, upon activation by pathogens, the expression of MARCH I is downregulated, leading to a restriction of the internal movement of MHC II within the cells [6,7,8,9,10].

The lymphocyte activation gene-3 (LAG-3) acts as a suppressive co-receptor found on T cells and is structurally similar to CD4, though it binds to MHC II with a higher affinity. LAG-3 expression is seen in activated CD4+ T cells, as well as in regulatory T cells, including Foxp3+ Tregs and Tr-1 cells [11]. When LAG-3 engages with MHC II, it hampers T cell activity through direct negative signaling involving the KIEELE motif on LAG-3 on the effector CD4+ T cells [12]. In the interaction between regulatory T cells and dendritic cells, LAG-3 engagement with MHC II dampens dendritic cell activation through the activation of the ERK pathway, which draws the SHIP-1 protein to the site [13]. Cells lacking cytoplasmic tails, when expressing LAG-3, can still exercise a regulatory influence, highlighting that LAG-3 and MHC II interaction serves as a two-way suppressive pathway. LAG-3 is crucial for the immunosuppressive capabilities of regulatory T cells and helps to restrain the inflammation by the macrophages residing in tissues [14]. Distinctly, LAG-3 differentiates MHC II molecular structures, binding selectively to stable peptide-MHC complexes rather than indiscriminately to all the peptide-MHC complexes [15]. Our studies demonstrate that LAG-3 associates with non-ubiquitinated MHC II, but not with MHC II that has been ubiquitinated.

In previous biophysical studies, we observed that the peptide/MHC complex I-Ak/HEL52-61, which binds peptides less tightly, displayed more pronounced rotational fluctuations when tracked using X-ray diffraction tracking (DXT) compared to the more stably bound peptide/MHC complex I-Ak/HEL48-61 [16]. We have since developed Diffracted X-ray Blinking (DXB), a novel technique used for single-molecule X-ray measurements [17]. Based on this, we theorized that the ubiquitination of the MHC could play a role in the regulation of T cell activation. Utilizing DXB, we have been able to monitor the dynamics of proteins on live cells in real time. Our findings suggest that the regulation of ubiquitinated MHC II may lead to various significant biological processes, such as the induction of regulatory T cells and the positive selection of CD4+ T cells.

## 2. Results

### 2.1. LAG-3 Preferentially Binds to Non-Ubiquitinated MHC II

**Figure 1:**
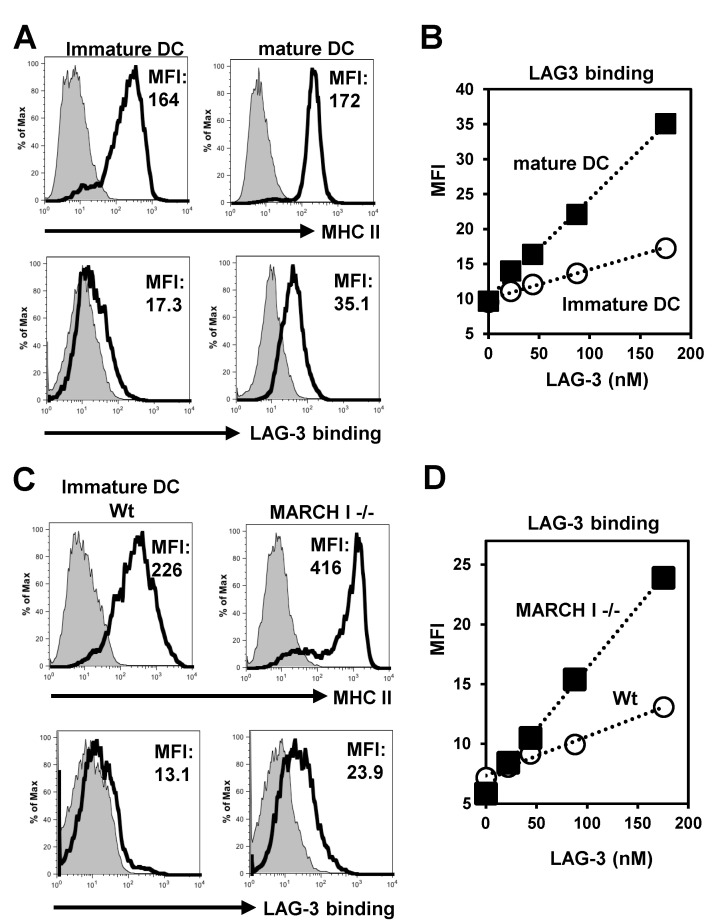
LAG-3 bound to non-ubiquitinated MHC II. (**A**) Flow cytometric analysis of MHC II expression and LAG-3 binding on CD11c-positive immature or mature DCs. (**B**) Binding of LAG-3 to immature or mature DCs. *X*-axis, input LAG-3 (nM); *Y*-axis, mean channel intensity (MFI). (**C**) Flow cytometric analysis of MHC II expression and LAG-3 binding on CD11c-positive wt immature DCs or MARCH I -/- immature DCs. (**D**) Binding of LAG-3 to wt immature DCs or MARCH I -/- immature DCs. Data are representative of three independent experiments.

In the previous reports, LAG-3 selectively binds to stable peptide-MHC complexes (pMHC) rather than indiscriminately to all MHC molecules [15]. Based on these results, we considered the possibility that LAG-3 may not attach to ubiquitinated forms of MHC II. To test this, we engineered soluble LAG-3 with a BirA biotinylation site using insect cells, which enabled us to form LAG-3 tetramers. According to Anderson et al. [11], these LAG-3 tetramers demonstrated stronger binding to the mature dendritic cells (DCs) than to the immature ones, as illustrated in Figure 1A. Additionally, we observed that the binding of LAG-3 tetramers to the mature DCs occurred in a dose-responsive manner (shown in Figure 1B). Since mature DCs have decreased expression of MARCH I, we hypothesized that LAG-3 may not associate with ubiquitinated MHC II.

Indeed, our experiments showed that LAG-3 had a high affinity for the immature dendritic cells (DCs) from the MARCH I deficient (-/-) mice, as depicted in Figure 1C. Furthermore, the binding of LAG-3 tetramers to these immature DCs occurred in a proportional relationship to the concentration, which is detailed in Figure 1D. These findings support the notion that LAG-3 does not interact well with ubiquitinated MHC II.

Indeed, our experiments showed that LAG-3 had a high affinity for the immature dendritic cells (DCs) from the MARCH I deficient mice, as depicted in Figure 1C. Furthermore, the binding of LAG-3 tetramers to these immature DCs occurred in a proportional relationship to the concentration, which is detailed in Figure 1D. We also examined the binding of LAG-3 Fc (a gift from Dr. Okazaki [15]) to the wt immature DCs, mature DCs, MARCH I deficient immature DCs, and mature DCs (Supplementary Figure S1) and obtained similar results. These findings support the notion that LAG-3 does not interact well with ubiquitinated MHC II.

**Figure 2:**
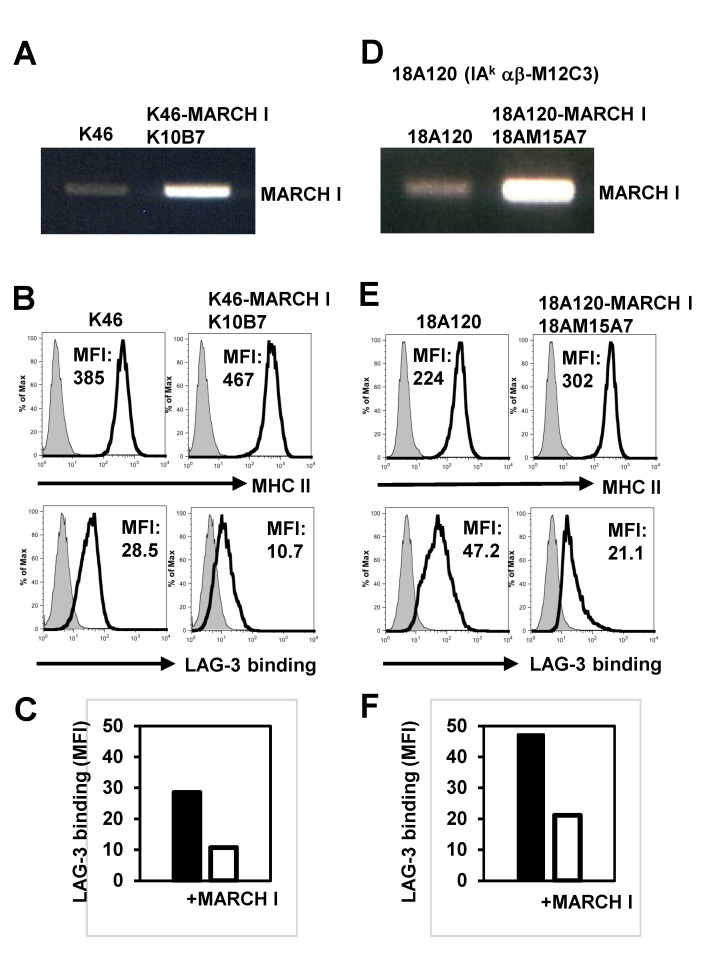
LAG-3 did not efficiently bind to ubiquitinated MHC II. (**A**) RT-PCR of MARCH I over-expressing K46 and K46, K10B7 (**B**) Flow cytometric analysis of MHC II expression and LAG-3 binding to K46 or K10B7. (**C**) Mean channel intensity (MFI) for binding of LAG-3 to K46 and K10B7 (**D**) RT-PCR of MARCH I against IAkαβ M12C3, 18A120 or MARCH I overexpression 18A120, M15A7. (**E**) Flow cytometric analysis of MHC II expression and LAG-3 binding to 18A120 and 18AM15A7. (**F**) MFI of LAG-3 binding to 18A120 and 18AM15A7.

In an effort to validate our theory, we engineered stable cell lines that expressed MARCH I, which would lead to the ubiquitination of MHC II. Despite high levels of MARCH I mRNA being present in both MARCH I-K46 (as shown in Figure 2A) and MARCH I-18A120 (M12C3-derived cell, as illustrated in Figure 2D), there was no observed decrease in the MHC II expression levels in MARCH I-K46 (Figure 2B) or MARCH I-18A120 (Figure 2E). However, the attachment of LAG-3 tetramers to both the MARCH I-K46 (Figure 2B) and MARCH I-18A120 (Figure 2E) variants was diminished. The Mean Fluorescence Intensity (MFI) of the LAG-3 tetramer binding to both MARCH I-K46 and MARCH I-18A120 is depicted in Figure 2C, F, respectively. This implies that LAG-3 interacts less effectively with ubiquitinated MHC II.

### 2.2. Ubiquitin Inhibits LAG-3 Binding to MHC II Conjugated Ubiquitin Number-Dependently

**Figure 3:**
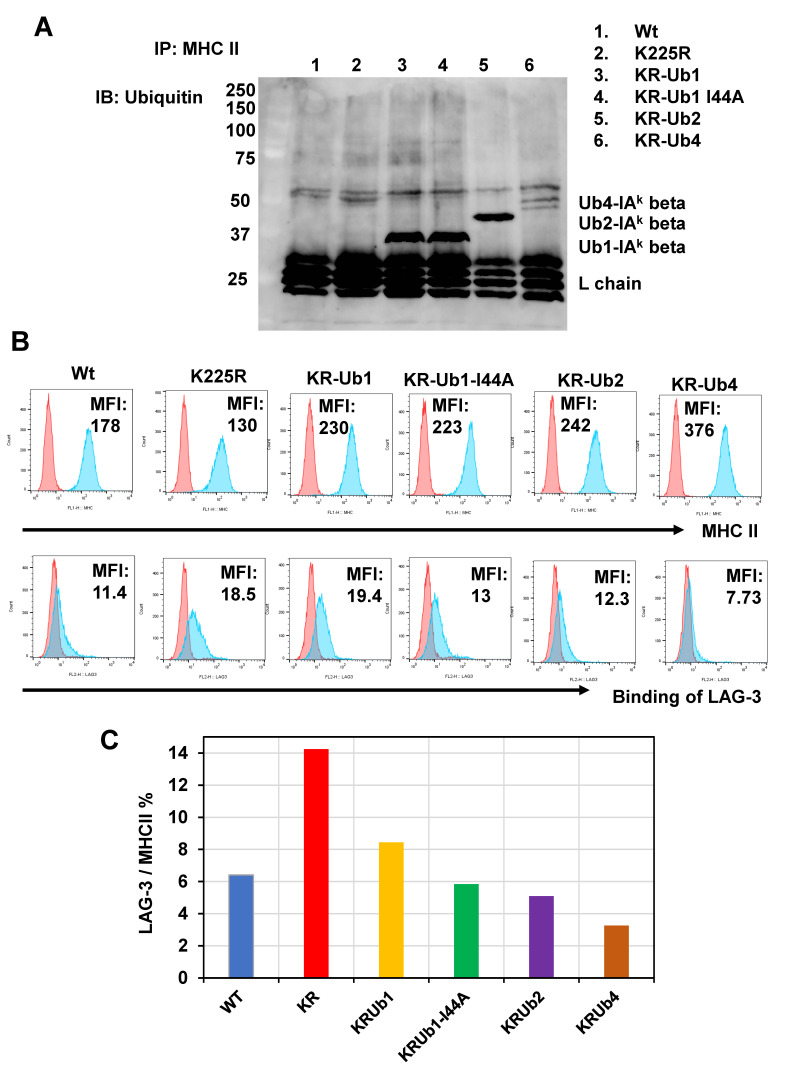
Binding of LAG-3 to MHC II decreased with increasing number of ubiquitin fixations. (**A**) Western blotting with anti-ubiquitin Ab P4D1 of IAk-wt, IAk-K225R, IAk-KR Ub1, IAk-KR Ub1-I44A, IAk-KR Ub2, and IAk-KR Ub4 after immunoprecipitation with anti-IAk α chain and β chain antibodies. Ubiquitin was not visualized in IAk-wt, whereas ubiquitin covalently bound to MHC was visualized in this condition. (**B**) Flow cytometric analysis of MHC II expression and LAG-3 binding in IAk-wt, IAk-K225R, IAk-KR Ub1, IAk-KR Ub1-I44A, IAk-KR Ub2, and IAk-KR Ub4. (**C**) Percentage of average channel fluorescence of MHC II expression in IAk-wt, IAk-K225R, IAk-KR Ub1, IAk-KR Ub1-I44A, IAk-KR Ub2, and IAk-KR Ub4 divided by the average channel fluorescence of LAG-3 binding.

We hypothesized that ubiquitination might influence the shape and robustness of the MHC II molecules. To explore this notion, we adopted a technique from Ira Melman’s group [18] to generate a set of ubiquitinated MHC II variants. Specifically, we engineered genetic sequences with ubiquitin irreversibly linked by substituting lysine (K) with arginine (R). Using M12C3 cells, which lack native MHC II expression, we produced stable cell lines containing IAk-wt, IAk-K225R, IAk-KR Ub1, IAk-KR Ub1-I44A, IAk-KR Ub2, and IAk-KR Ub4 (Supplementary Figure S2). Immunoprecipitation with MHC II revealed ubiquitin bands in IAk-KR Ub1, IAk-KR Ub1-I44A, IAk-KR Ub2, and IAk-KR Ub4, as indicated in Figure 3A. Despite similar expression levels of MHC II, the binding of the soluble LAG-3 (sLAG-3) tetramer varied with the number of ubiquitin molecules, showing a decrease, as depicted in Figure 3B. The Mean Fluorescence Intensity (MFI) ratio of LAG-3 tetramer binding to MHC II expression, shown in Figure 3C, indicated that ubiquitin inhibited LAG-3 binding in a ubiquitin number-dependent manner. Therefore, ubiquitination seems to modify the structure and stability of MHC II.

### 2.3. DXB Reveals Increased Dynamics of Ubiquitinated MHC II

We recently implemented Diffracted X-ray Blinking (DXB) [17], a technique used for measuring single molecules with a laboratory X-ray setup. A novel DXB application was developed to study the surface of cells. Using this approach, either I-Akαβ+ or I-Akαβ K225R+, MARCHI-transfected M12C3 cells were affixed to Kapton paper alongside anti-IAk antibody-coated gold nanocrystals, followed by X-ray exposure. During the low-dose, monochromatic X-ray diffraction experiments, the intensity of the diffraction spots from the nanocrystals fluctuated due to Brownian motion in the liquid environment. The periodic movement of the diffraction spots, in and out of the Bragg condition, was noted and captured using a CCD camera. The time-dependent intensity fluctuations of individual pixels were analyzed with an autocorrelation function (ACF), linking the ACF decay constant to the rotational speed of the gold nanocrystals. Here, we conducted DXB in the presence of 0.1% Sodium Azide, that decreased the intracellular ATP levels and inhibited energy-dependent endocytosis. In other words, the effect of MHC II endocytosis due to ubiquitination is negligible.

**Figure 4:**
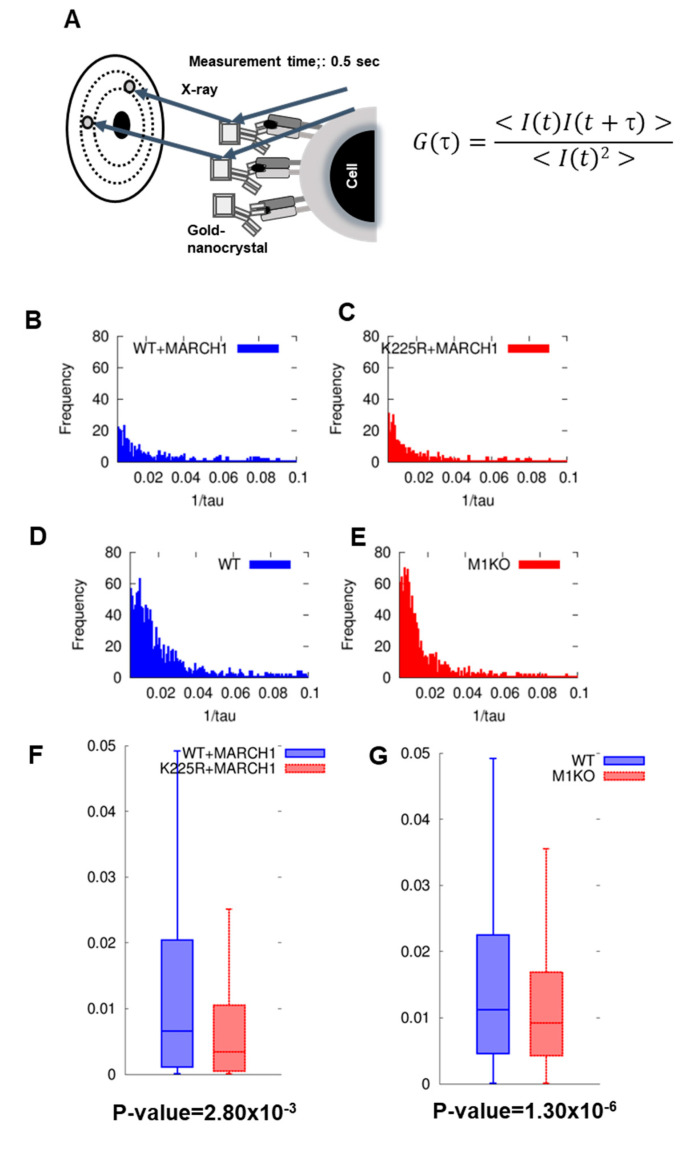
The movement of ubiquitinated MHC II detected on cells by Diffracted X-ray Blinking (DXB) was faster than that of non-ubiquitinated MHC II. (**A**) Schematic of Diffracted X-ray Blinking (DXB). Gold nanocrystals were bound to MHC II antibodies. After incubation of the nanocrystals and cells, white X-rays were irradiated, and the diffracted blinking spots were videotaped. (**B**) Attenuation constant distribution of IAkαβ + MARCH I-transfected M12C3. (**C**) Attenuation constant distribution of IAkαβ K225R + MARCH I-transfected M12C3 (**D**) Attenuation constant distribution of wt immature DC. (**E**) Attenuation constant distribution of MARCH I deficient immature DC. (**F**) Comparison of attenuation constant distributions using box-and-whisker plots of wt + MARCH I (blue) and K225R + MARCH I (red). The median attenuation constant was 6.75 × 10−3 for wt + MARCH I and 3.25 × 10−3 for K225R + MARCH I. (**G**) Comparison of attenuation constant distributions for wt immature DC (blue) and MARCH I deficient immature DC (red). Median attenuation constants were 11.15 × 10−3 for wt immature DCs and 9.65 × 10−3 for MARCH I deficient immature DCs. *p*-value = 1.30 × 10−6.

Figure 4A depicts the DXB imagery, while Figure 4B–E categorizes the ACF decay constant frequencies. Figure 4F compares the ACF decay constant distributions for the ubiquitinated I-Akαβ + MARCH I-M12C3 and non-ubiquitinated I-Akαβ K225R + MARCH I-M12C3 cells, showing median decay constants of 6.75 × 10^−3^ s^−1^ and 3.25 × 10^−3^ s^−1^, respectively. 

A non-parametric Wilcoxon rank sum test showed a significant difference in the ACF decay constants between the wild-type and the K225R mutant I-Akαβ (*p*-value = 2.80 × 10^−11^). According to Table 1, the I-Akαβ + MARCH I-M12C3 cells exhibited increased sensitivity, with higher diffraction spot movement, rotational speed, and attenuation constant compared to those of I-AkαβK225R + MARCH I-M12C3. This indicates that ubiquitinated MHC II may possess increased mobility compared to its non-ubiquitinated counterpart.

Figure 4G presents the comparative decay constant distributions for immature dendritic cells (DCs) in both the wild-type (wt) and MARCH I-deficient (MARCH I -/-) mice. The immature DCs from the wt mice, which had ubiquitinated MHC II, showed higher decay constants than those from the MARCH I deficient mice, which lacked ubiquitinated MHC II. Specifically, the median values of decay constants were 11.15 × 10^−3^ s^−1^ for the wt immature DCs and 9.65 × 10^−3^ s^−1^ for the MARCH I deficient immature DCs. Notably, the decay constant distribution in the wild type was significantly different from the MARCH I knockout (M1KO), with a *p*-value of 1.30 × 10^−6^. Table 1 outlines that not only the median values of decay constants, but also the velocity, angular velocity, and distance measurements for diffraction spot movements were marginally higher in the wt immature DCs compared to the MARCH I deficient ones. This implies that ubiquitinated MHC II may exhibit a more rapid response when analyzed using DXB, as opposed to non-ubiquitinated MHC II.

## 3. Discussion

Our research revealed that the interaction of ubiquitinated MHC II with the LAG-3 tetramer, as well as the application of DXB, affected its structure and stability; there was a notable increase in the affinity of the LAG-3 tetramer for the mature dendritic cells (DCs) compared to that of the immature DCs. Furthermore, the LAG-3 tetramer exhibited a stronger connection with the immature DCs deficient in the ubiquitin ligase MARCH I than with those of the wild type (wt). The presence of MARCH I in the transfected cells correspondingly diminished their interaction with the LAG-3 tetramer, similarly to the targeted binding between ubiquitin and MHC II. Ultimately, our findings indicate that the ubiquitination of MHC II influences the structural conformation and stability, as detected by the binding efficacy of LAG-3.

The ubiquitination of MHC II is complicated by the involvement of multiple ubiquitin ligases; not only is MHC II itself subject to ubiquitination, but the related molecules DM and DO are also targets for MARCH I, MARCH VIII, and MARCH IX [19,20]. While MARCH I is implicated in MHC II’s intracellular transport, DM’s localization to lysosomes is directed by the lysosomal targeting signal sequence YTPL [21]. DO associates with DM within the lysosomes [22]. This YTPL sequence may trigger ubiquitin ligases for lysosomal localization. 

CD83 is a known marker of mature DCs. However, the CD83-deficient mice were specifically inhibited in the development of CD4+ monocyte-positive thymocytes [23]. The transmembrane domain of CD83 on the mature DCs enhanced MHC II expression by inhibiting the interaction between MHC II and MARCH I [24] and inhibited the MARCH I-dependent ubiquitination and degradation of MHC II by IL-10. The transmembrane domain of CD83 is necessary and sufficient for CD4 T cell selection [25]; the ablation of MARCH VIII in the CD83-deficient mice restored CD4 T cell development [25]. Thus, ubiquitinated MHC II on thymic epithelial cells may regulate positive selection via CD83.

We recently enhanced the Diffracted X-ray Blinking (DXB) technique for studying how soluble acetylcholine-binding proteins interact with acetylcholine using low-dose monochromatic X-rays in conjunction with nanocrystal labeling, and oligomers of the amyloid β isoforms [17,26]. Applying this advanced DXB, we investigated the MHC II on cellular surfaces. Through DXB, we observed that the ubiquitinated MHC II molecules demonstrated quicker movements than their non-ubiquitinated counterparts. The previous research by Roy et al. identified a disruption in cholesterol binding within the transmembrane region of MHC II [27], along with a cholesterol reduction during Leishmania infection that modulates the immune responses [28]. Consequently, it is plausible that the ubiquitination of MHC II may similarly influence the structural dynamics of its extracellular domain.

Therefore, the ubiquitination of MHC II not only promotes degradation and relocation of the protein from the cell membrane to the interior of the cell, but also changes its dynamic structure. This subtle conformational change makes the structure detectable by both TCR and LAG-3.

## 4. Materials and Methods

### 4.1. Mice and Cells

MARCH I-deficient mice (CBA background, I-Ak) [5] were transferred to the pathogen-free animal breeding facility at the Tokyo University of Science, Research Institute for Biomedical Science. All experiments were performed according to protocols approved by the Animal Welfare Committee of the Tokyo University of Science.

Bone marrow cells were harvested from the femur and tibia of the mice. The cells were carefully pipetted and suspended. Erythrocytes were lysed by ACK treatment for 1 min at room temperature. After washing, the cells were passed through 100 μm nylon mesh to remove debris. The cells (1.6–2 × 10^6^)/10 mL were incubated with 10% FCS, 2 mM glutamine, 10 mM HEPES, 1% penicillin/streptomycin, 1 mM sodium pyruvate, 1% non-essential amino acids, 50 μM β-mercapto-ethanol (complete medium), and 20 ng/mL recombinant GM-CSF (kindly donated by Dr. Abe of the same institute) and cultured in a 5% CO_2_ incubator for 10–11 days to differentiate into immature DCs. On days 3, 8, and 10, fresh medium containing the same amount of cytokine was added. To create mature DCs, 1 μg/mL LPS (*E. coli* 055B5, Sigma-Aldrich, Waltham, MA, USA) was added on day 9 or 10.

### 4.2. Reagents

Anti-I-Akβ (clones 10–3.6), anti-I-Akα (clone 11.5.2), anti-CD11c (clone N418), anti-CD86 (clone GL-1), and control mouse IgG2a (clone MG2a-53) were purchased from BioLegend (San Diego, CA, USA). Purified CD16/CD32 (clone 2.4G2) was purchased from BD Pharmingen (Flanklin Lakes, NJ, USA). Streptavidin-PE was purchased from Thermo Fisher Scientific (Waltham, MA, USA).

Anti-ubiquitin (P4D1) was purchased from BioLegend; HRP-anti-mouse κ was purchased from Bethyl (Hamburg, Germany); Western Lightning ECL Pro (NEL 120001) was purchased from PekinElmer (Waltham, MA, USA). Chemiluminescence signals were analyzed with an LAS3000 (FUJIFILM, Tokyo, Japan).

### 4.3. Flow Cytometry 

Single-cell suspensions of DC or tissue culture cells were blocked and stained with cell surface markers or mouse IgG2a isotype control in 1% BSA-PBS-0.1% Sodium Azide (FACS buffer) on ice in the dark for 60 min. The cells were washed and analyzed using an FACSCalibur cytometer (BD biosciences, Flanklin Lakes, NJ, USA). Data analysis was performed using FlowJo software Ver 8.2 and Ver 10.8.1(Flow jo, LLC Ashland, OR, USA).

### 4.4. Generation of MHC II- and MARCHI-Expressing Cells

I-Akα and β strands were amplified from the c-DNA of a CBA spleen reverse-transcribed with PrimeScript II 1st strand synthesis kit (Takara, Kusatsu, Shiga, Japan) and cloned at the GFP site and the multicloning site, respectively, into the K225R mutant of the I-Ak β strand induced by a PCR-based site-directed mutagenesis method. pEF1 alpha was cloned into pEF1 alpha (Takara). Ubiquitinated MHC II was cloned using artificial DNA (Eurofins genomics, Tokyo, Japan), with 7 lysines replaced with arginine and C-terminal glycine replaced with valine and combined with the K225R mutant of the I-Ak b strand [18]. M12C3 cells which did not express MHC II [29] were transfected with I-Ak α chain and β chain, I-Ak α chain, and K225R β chain, or ubiquitinated I-Ak α chain and K225 R β chain using The Gene Pulser (BioRad Laboratory Inc., Hercules, CA, USA) and selected in 96-well plates using 0.3 mg/mL G418 (Thermo Fisher Scientific, Waltham, MA, USA). The expression of IAkα and IAkβ was detected with FITC-anti-IAk α (11.5.2, BioLegend) or PE-anti-I-Ak β (10.3.6, BioLegend). Using M12C3 cells, a certain number of ubiquitinated MHCs were generated according to the method of Ira Melman group [18] to produce IAk-KR Ub1, IAk-KR Ub1-I44A, IAk-KR Ub2, and IAk-KR Ub4. The K was replaced by R. That is, K was replaced by R to create a gene sequence with covalently bound ubiquitin. Expression clones of the I-Ak α and β chains and both the 18A120 and I-Ak α chains were used in pSP-MARCH I-puro (pSp Cas9 (BB)-2a-puro (PX459) Addgene replaced Cas9 with MARCH I amplified from pEF MARCH I [5]) and transfected in 96 well plates at 1.2 μg/mL. The selection was made using puromycin; RNA was extracted from single-colony clones using TRizol (Thermo Fisher Scientific, Waltham, MA) and reverse-transcribed using the PrimeScript II 1st strand synthesis kit (Takara). Short MARCH I PCR products were amplified using 5′ (TGTATGTGTTGATAGATCGGGAC) and 3′ (GACTGGTATAACCTCAGGTG).

K46 cells (B-cell lymphoma) were also transfected with pSP-MARCH 1-puro and selected using 1 μg/mL puromycin in 96-well plates.

### 4.5. LAG-3 Tetramers

The ectodomain of LAG-3 amplified by RT-PCR from CBA spleen c-DNA was cloned into pFast BacTM Dual (Thermo Fisher Scientific), with BirA sites and FLAG tags added. The cells were infected with SF9 Invitrogen and cultured in SF900 II serum-free medium (Thermo Fisher Scientific) at 18 °C for 6–7 days. Protein-containing culture supernatants were adjusted to pH 7.0 and loaded onto M2 column (Sigma-Adrich, Waltham, MA, USA), proteins were eluted with 100 μM FLAG peptide in PBS. Isolated LAG-3 was biotinylated with the BirA Biotin-Protein ligase kit (BirA 500, Avidity, LLC, Hercules, CO, USA), followed by the removal of excess biotin with Micro Bio-Spin chromatography columns (BioRad). Biotinylated LAG-3 was mixed with streptavidin PE (Agilent Technology, Santa Clara, CA, USA) to form LAG-3 tetramers.

### 4.6. Diffracted X-ray Blinking

Gold nanocrystals were prepared through epitaxial growth on cleaved KCl(100) (area: 7 × 7 mm^2^) under a vacuum of 10^−4^ Pa as previously described [17]. The shape and quality of the gold nanocrystals were confirmed using atomic force microscopy for 1000 particles in a 100 mm^2^ NaCl substrate. Anti-I-Ak β (10.3.6, BioLegend) antibodies or control IgG2a were bound to the gold nanocrystals via cysteine. The cells blocked with anti-CD16/CD32 were immobilized on 50 mm thick Kapton Polyimide film (DuPont-Toray, Tokyo, Japan) via poly-L-lysine (Sigma-Aldrich). Antibody-attached gold nanocrystals were incubated with the cells on polyimide film in PBS-0.1% Sodium Azide at 277K. Laboratory DXB measurements were performed using a laboratory X-ray source (Rigaku FR-D: Cu anode 50 kV, 60 mA), and time-resolved diffraction images were recorded using a 2D photon counting detector (Pilatus 100 K, Dectris, Villigen, Aargau, Switzerland). The distance from the sample to the detector was 30 mm, the exposure time per frame was 1.0 s, and the interval time was 1.003 s. From each pixel around the Au(111) diffraction ring, the intensity trajectory was extracted, except between the rectangular regions of the Pilatus detector. The data were analyzed in C and Gnuplot on the Linux platform. The time course of the diffracted light signal I(t) provided information about the motion of the probe particles. The fluctuations in this signal are due to changes in the diffraction yield (number of photons per particle per second) of the particles in the open probe volume, defined by the focal volume of the X-ray beam. To analyze these fluctuations, the autocorrelation function (ACF) of the photon intensity can be calculated as follows.

$$ACF=\frac{<I\left(t\right)I\left(t+\tau \right)>}{<I{\left(t\right)}^{2}>}$$

where the square brackets indicate the time average, I(t) is the number of diffraction pictures as a function of time, and τ is the delay time; the ACF is processed for each pixel on the Au(111) diffraction ring, except in the detector-intermodular spaces, and in the range during the first 10% of the observation time, a single exponential curve, ACF = k + A exp(-Tt), was fitted, where k is a constant, A is the amplitude, and T = 1/τ is the decay constant. The distribution of decay constants among the different cells was visualized using box-and-whisker plots and was compared using the non-parametric Wilcoxon rank sum test. The decay constant (s^−1^) represents the time it takes for the diffraction intensity to decrease by 63%, indicating the movement of diffraction spots between pixels. The velocity (μm s^−1^) was calculated as 1 pixel distance (μm) × (1.37 × decay constant (s^−1^). The angular velocity (mrad s^−1^) was calculated as tan^−1^ (distance travelled in diffraction spot/distance between camera and gold nanocrystal) (rad) × decay constant (s^−1^).

## Figures and Tables

**Table 1:** DXB summary data.

	WT + MARCH I	K225R + MARCH I	WT DC	M1KO DC
Attenuation constant (s^−1^)	6.75 × 10^−3^	3.25 × 10^−3^	12.15 × 10^−3^	9.65 × 10^−3^
Verocity of dffraction spots (μm s^−1^)	0.73	0.35	1.2	1.04
Angular verosity (mrad s^−1^)	1.4	0.67	2.3	2
Distance (Å s^−1^)	0.38	0.182	0.62	0.54

Ubiquitinated MHC II showed greater mobility than non-ubiquitinated MHC II.

## Data Availability

Data is contained within the article and Supplementary Materials.

## Supplementary Materials

The following supporting information can be downloaded at: https://www.mdpi.com/article/10.3390/ijms242317083/s1.
