# Supplementary material for: Ubiquitination of Major Histocompatibility Complex II Changes Its Immunological Recognition Structure
Source: Int J Mol Sci. 2023 Dec 3;24(23):17083. doi: 10.3390/ijms242317083 (PMC10707457; doi:10.3390/ijms242317083)
Supplement: Supplementary file 1 [file ijms-24-17083-s001.zip › ijms-2717191-supplementary.pdf]

Supplementary Figures

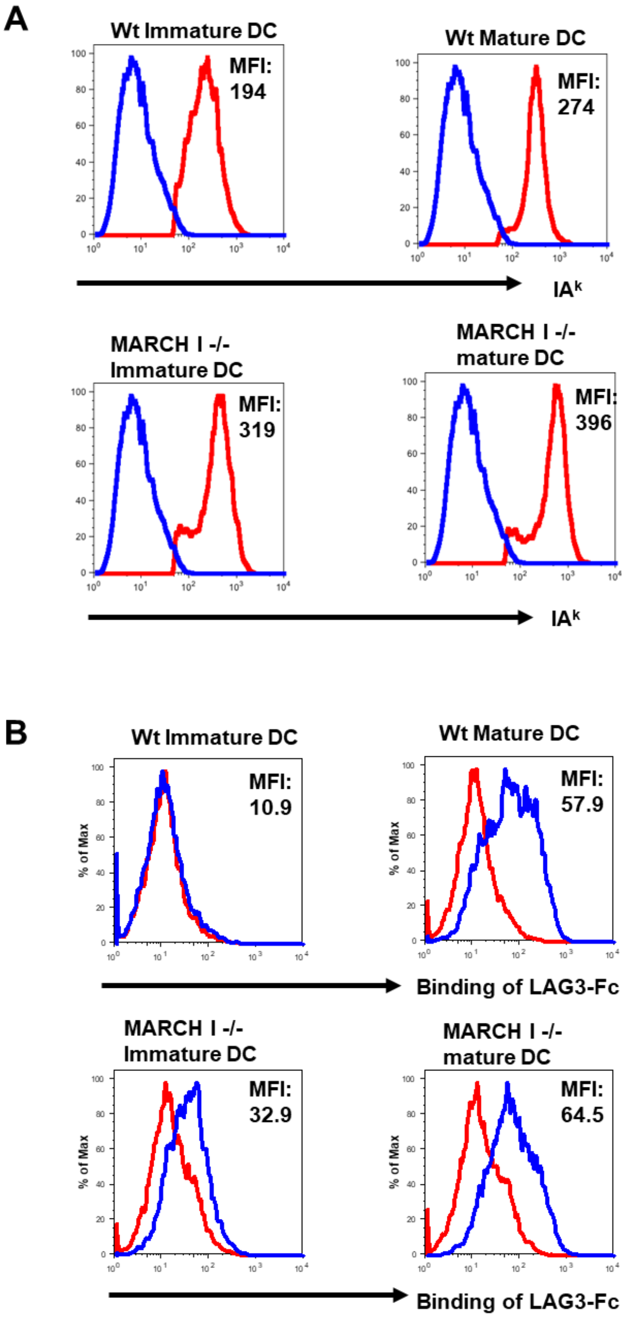

Supplementary Fgure S1. LAG-3 bound non-ubiquitinated MHC II. Immature DCs were differentiated from either wt bone marrow cells or MARCH I -/- bone marrow cells in the presence of GM-CF for 10 days. Mature DCs were stimulated by LPS for last 24 h. (A) Flow cytometric analysis for expression of MHC II on CD11c positive immature DCs or Mature DC. (B) Flow cytometry analysis for binding LAG-3 Fc (gift from Dr. Okazaki from Tokushima University) on CD11c positive immature DC or Mature DC.

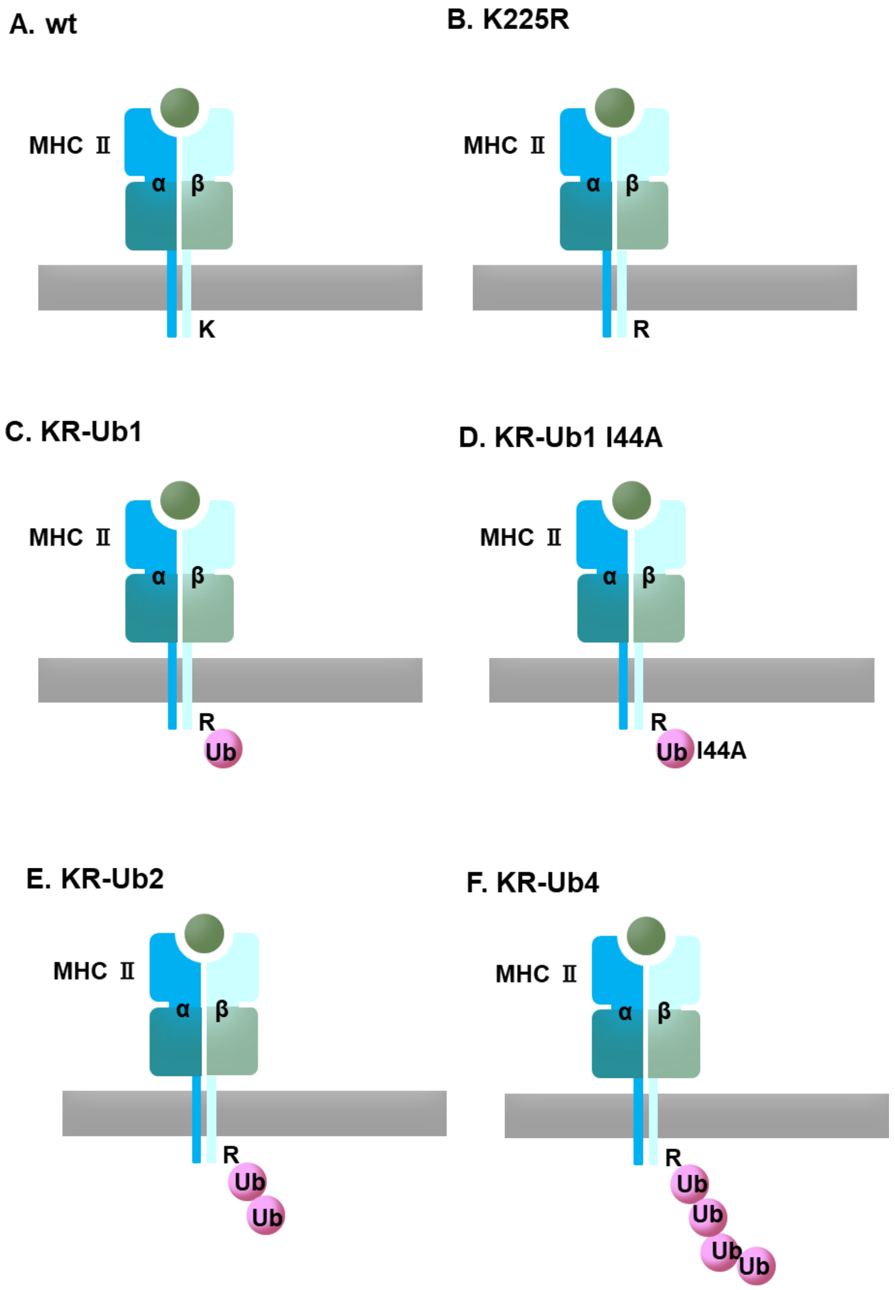

Supplementary Figure S2. Stable transfectant for IAkαβ with CLIP. (A) wt IAkαβ. (B) K225R mutation of IAkβ chain. (C) A ubiquitin was covalently linked to K225R mutation of IAkβ chain. (D) A ubiquitin that was induced I44A which is binding site of ubiquitin was covalently linked to K225R mutation of IAkβ chain. (E) Two ubiquitins were covalently linked to K225R mutation of IAkβ chain. (F) Four ubiquitins were covalently linked to K225R mutation of IAkβ chain.
